# Possible sinoatrial node dysfunction in a 6-month-old domestic shorthair cat

**DOI:** 10.1177/20551169241291841

**Published:** 2024-12-06

**Authors:** Florence Hillen, Laurent Locquet

**Affiliations:** 1 School of Veterinary Medicine and Science, University of Nottingham, Sutton Bonington Campus, Leicestershire, UK; 2 Dick White Referrals, Station Farm, London Road, Six Mile Bottom, Cambridgeshire, UK

**Keywords:** Sinoatrial node dysfunction, pre-anaesthetic assessment, cardiology, ambulatory electrocardiogram

## Abstract

**Case summary:**

A 6-month-old male entire domestic shorthair cat was presented to the ophthalmology department for nasolacrimal duct cannulation and flushing, and castration under general anaesthesia. On pre-anaesthetic assessment, the cat had a heart rate of 90 beats/min (bpm). Clinical examination was unremarkable, although the cat appeared stressed. The echocardiogram was within normal limits. An ambulatory electrocardiogram (ECG) monitor was fitted overnight, and analysis of the ECG revealed a sinus rhythm with a lower than normal heart rate. The mean 1 min rate was 98 bpm. There was a slower than normal sinus rhythm and frequent ventricular escape beats. Differential diagnoses included increased vagal tone and sinoatrial node dysfunction (SND). The latter was suspected as the cat demonstrated signs of stress although an atropine response test was not performed.

**Relevant and novel information:**

SND is relatively common in dogs but extremely rare in cats. To the authors’ knowledge, there is only one affected cat mentioned in the literature. In both dogs and humans, most cases described are in middle-aged and elderly patients. Although the condition is recognised in human infants and fetuses, it has not been reported in dogs under 2 years of age. This case is unusual because SND was suspected in an immature cat. On analysis of the ECG, the heart rate was considerably lower than those previously reported in hospitalised cats; however, findings on physical examination were subtle, highlighting the importance of pre-anaesthetic examination in identifying unexpected abnormalities.

## Introduction

Sinoatrial node dysfunction (SND) and sick sinus syndrome (SSS) are relatively common in dogs^1–15^ but rare in cats. This case describes the incidental finding of a bradyarrhythmia, possibly SND, during a pre-anaesthetic assessment of a 6-month-old domestic shorthair cat. To the authors’ knowledge, SND has not been reported in cats or in juvenile animals.

## Case presentation

A 6-month-old male entire domestic shorthair cat was presented to the ophthalmology department for nasolacrimal duct cannulation and flushing, and castration under general anaesthesia. A presumptive diagnosis of feline upper respiratory tract infection was made by the referring veterinary surgeon after epiphora and clinical signs in littermates. The aetiology was not identified and the cat was prescribed doxycycline 10 mg/kg PO q24h, topical chloramphenicol 1% and sodium hyaluronate 1.4% q12h, and 0.05 mg/kg meloxicam PO q24h. Upon admission to the hospital, the owner reported intermittent sneezing, respiratory stertor and episodic panting.

On initial physical examination, the cat had a heart rate of 140 beats/min (bpm), a capillary refill time of <2 s, synchronous peripheral pulses and a respiratory rate within normal limits. Thoracic auscultation was unremarkable. On pre-anaesthetic assessment by a veterinary nurse (RVN), a heart rate of 90 bpm was recorded. Signs of stress were noted. The patient was referred to the cardiology department. A multiparameter monitor (uMEC12 Vet; Mindray) was used for continuous monitoring of the electrocardiogram (ECG) before assessment by a cardiologist. A heart rate of 33–80 bpm was observed with no abnormal waveform complexes; however, heart rates <68 bpm could not be confirmed by auscultation and mis-sensing by the monitor was suspected.

Complete blood count (CBC) and serum biochemistry were unremarkable. Feline coronavirus serum antibody ELISA was negative and alpha1-acid glycoprotein was within normal limits (<300 µg/ml). Feline immunodeficiency virus antibody and feline leukaemia virus antigen (SNAP FeLV/FIV Combo; IDEXX) tests were negative, as was the test for *Toxoplasma* species antibodies (IgG and IgM).

**Figure 1:**
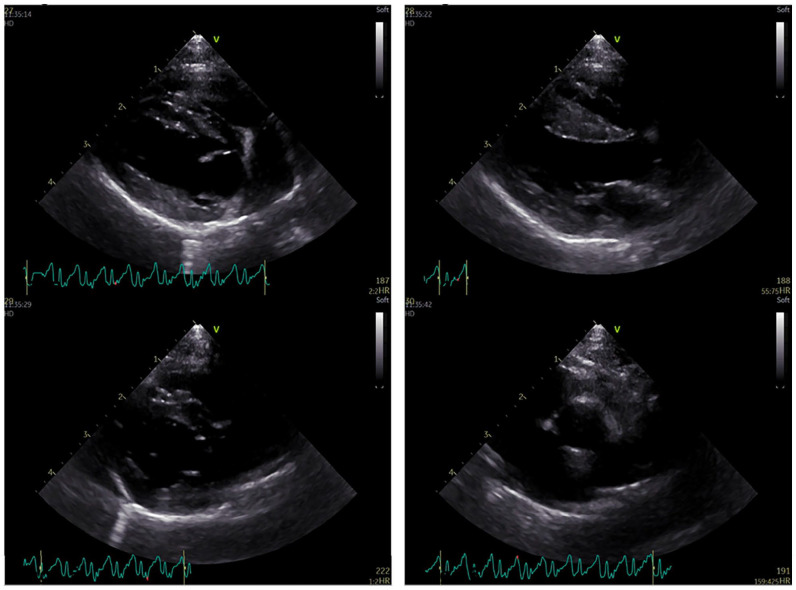
Two-dimensional echocardiogram showing the right parasternal long-axis and short-axis views demonstrating normal atrial and ventricular size, wall thickness and mitral valve morphology. The left atrial:aortic ratio is within normal limits (1:1.3)

Echocardiography (Vivid E95 Ultra Edition; GE Healthcare), including M-mode, two-dimensional and Doppler studies, was unremarkable (Figure 1). The ECG recorded a sinus rhythm.

**Figure 2:**
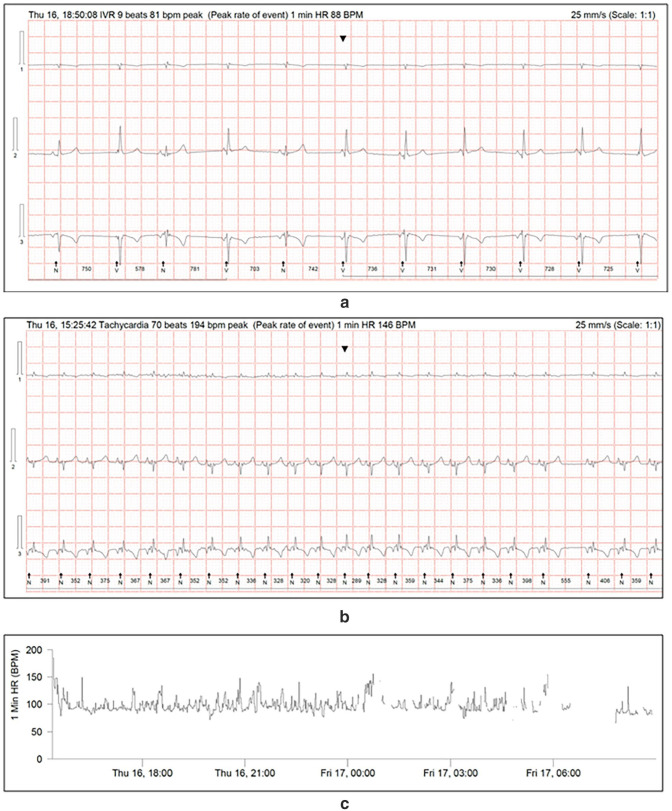
(a) Electrocardiogram (ECG) obtained from the ambulatory ECG monitor showing episodes of idioventricular rhythm, (b) a slower than normal sinus rhythm at the peak rate for this episode and (c) the tachogram with areas of baseline artefact removed

The cat was hospitalised and an ambulatory ECG monitor was fitted for overnight ECG recording (Lifecard CF; Spacelabs Healthcare). An analysis revealed a sinus rhythm with a heart rate lower than normal. This was despite demonstrable signs of stress while kennelled. The mean 1 min rate was 98 bpm (range 74–149). There was a sinus rate lower than normal, with frequent ventricular escape beats (VEBs) totalling 1139 complexes over a period of 17 h and 36 mins, including periods of idioventricular rhythm. A total of 229 episodes consistent with sinus rhythm at a rate considered normal were noted with a maximum 1 min average rate of 149 bpm, an instantaneous rate of 194 bpm and a maximum duration of 366 beats (Figure 2).

Treatment was not necessary and the owner declined to proceed with the general anaesthesia for elective castration. The owner was advised to disallow outdoor access to prevent unwanted mating. Conscious cannulation and flushing of the nasolacrimal duct were not possible and medical management of epiphora was continued.

On re-examination 4 months later, the cat had a heart rate of 132 bpm with a regular rhythm. Physical examination was otherwise unremarkable. Echocardiography was within the normal limits and the ECG revealed sinus bradycardia. The owner reported no concerns except for persistent mild upper respiratory tract stertor, likely resulting from chronic tissue fibrosis.

## Discussion

Heart rates in healthy unrestrained hospitalised cats are reportedly in the range of 128–256 bpm,^16^ with means of 150 ± 23 bpm increasing to 187 ± 25 bpm during handling.^17^ Lower rates are found in cats in their home environment, in the range of 110–250 bpm,^16^ 68–294 bpm^18^ and 70–303 bpm^19^ with means of 132 ± 19 bpm^17^ and 157 ± 3.7 bpm.^18^ Sinus arrhythmia is abnormal in hospitalised cats but is common at home.^17–19^ Cats are prone to stress during hospitalisation and increased sympathetic tone is probably responsible for these differences. Our cat had a mean 1 min rate of 98 bpm and a minimum of 74 bpm despite being hospitalised. A rate of 140 bpm at presentation was considered normal, explaining why bradycardia was not initially recognised. Rates this low are uncommon in stressed animals and this perhaps should have been considered abnormal. On re-evaluation, a rate of 90 bpm was auscultated. This was identified by an RVN, highlighting their invaluable role in the monitoring, evaluation and management of patients.

The diagnosis of arrhythmias typically requires ECG analysis. Arrhythmias may not be continuously present and an ambulatory ECG monitor, or cardiac event recorder, is occasionally necessary to achieve diagnosis.^20^ Echocardiography is useful to identify structural causes, and serum biochemistry and CBC to eliminate underlying inflammatory disease and electrolyte disturbances.^2^ Obtaining a thorough clinical history is important to rule out medication or toxicity as potential causes.^21^

In the present cat, echocardiography, serum biochemistry and CBC were within the normal limits. An ECG analysis revealed an underlying sinus rhythm with 1139 VEBs identified, including periods of idioventricular rhythm. Ventricular ectopic beats occur commonly in clinically normal cats but less frequently. Over a 24 h period, Ware^18^ identified ranges of 0–59 episodes in all but one cat, which had 729 episodes and suspected enhanced ventricular automaticity,^18^ while Hanås et al^19^ found ranges of 0–146 complexes with an average of three.^19^ In the present patient, significantly more complexes were identified. Possible explanations include elevated vagal tone, myocarditis affecting the sinoatrial node (SAN) or surrounding tissue, and SND.

Sinus arrhythmia and sinus bradycardia are less common in cats than in dogs owing to relatively increased sympathetic drive. Elevated vagal tone can be associated with intracranial lesions and pathology of the abdomen, eye and respiratory tract.^22^ The upper respiratory tract signs in the present cat may have been responsible for parasympathetic predominance although the signs of stress raised the suspicion of SND.

SND and SSS are relatively common in humans^23^ and dogs^1–13,15,24^ but rare in cats. To the authors’ knowledge, there is one mention of a cat with SSS in the literature included in a retrospective analysis of outcomes after implantation of an epicardial pacemaker.^25^ In dogs, most cases are in middle-aged or elderly animals with females and some breeds overrepresented.^1,3,15,24^ There are occasional reports in dogs aged as young as 2 years.^1,2^ In humans, most cases are reported in elderly patients^23^ and only occasionally in fetuses, infants and children.^26^

This case is unusual because a low heart rate was detected in an apparently stressed cat although a definitive diagnosis of SND was not made because excessive vagal tone was not excluded. In humans, electrophysiological testing can be used to confirm a diagnosis,^3^ but is rarely performed in animals owing to requirements for specialist equipment. An atropine response test was warranted to distinguish between intrinsic disease of the SAN and vagally mediated arrhythmias, although the response in dogs is variable.^3^ The test was declined because the patient was asymptomatic and the owner elected not to proceed with surgery.

The ECG findings were consistent with reports of SND in dogs and humans where sinus bradycardia or arrest is terminated by VEBs.^1,3,21^ Electrocardiographic findings in affected patients include sinus bradycardia, episodic sinus arrest, second degree atrioventricular block, VEBs, periods of idioventricular rhythm, paroxysmal supraventricular tachycardia and atrial fibrillation.^27^ Junctional or ventricular escape rhythms occur in response to inadequate rates of SAN depolarisation. In the current patient, frequent VEBs and periods of sequential escape complexes were reported.

SND and SSS are characterised by defects in automaticity or conduction of the SAN, resulting in heart rates inadequate to maintain normal physiological function. Patients present without clinical signs if the rate produced by alternative pacemaker cells is sufficient to maintain appropriate cardiac output. Clinically affected individuals develop arrhythmias leading to syncopal episodes, exercise intolerance, weakness and lethargy.^1,3^ Nomenclature in the literature is inconsistent, but SND generally refers to abnormal conduction in an asymptomatic patient, while SSS describes clinical disease.^1^ In this case, episodes of panting were reported, but these were likely to be associated with upper respiratory tract obstruction and were not associated with syncope. It is possible that clinical signs in cats may be observed only with more advanced disease when compared with dogs owing to their increased tendency to modify activity to accommodate pathology.

Most cases of SSS in humans and dogs are idiopathic.^21,28^ Fibrosis of nodal tissue in humans^21^ and fibrous or fibro-fatty infiltration in dogs preventing myocardial conduction is described.^24,29^ Similar histopathological findings have been identified in dogs with mitral and tricuspid valve regurgitation.^24^ Less common causes in humans include myocardial infiltrative disease,^30^ including neoplasia,^31^ viral destruction of the SAN and central autonomic dysfunction.^32–35^ Congenital mutations of the alpha-subunit of the cardiac sodium channel (*SCN5A*) may explain cases identified in fetuses and children.^26^ In dogs, breed disposition suggests a genetic basis.^1,3,24^ Because of the age of the present cat, congenital disease was considered likely.

Despite the history of suspected viral rhinitis, viral myocarditis was thought unlikely. Viral destruction of sinoatrial pacemaker cells is reported in humans with COVID-19 infection^33–35^ but is not described in domestic animals. The aetiology of the rhinitis was not identified but common causes are feline calicivirus (FCV) and feline herpesvirus-I (FHV-1).^36^ Neither are known to cause myocarditis and neither have been identified on PCR of formalin-fixed hearts.^37^ Herpes simplex virus encephalitis has been implicated in central autonomic dysfunction in humans depressing SAN depolarisation.^32^ FHV-1 and FCV are reported to cause encephalitis in cats;^38^ however, in this case, there was no central nervous system involvement. Myocardial infiltrative disease has been reported in cats with lymphoma^39–41^ and hyper-eosinophilic syndrome.^42^ A cardiac troponin assay was warranted to rule out myocardial injury although this was considered unlikely given normal echocardiography findings.

Treatment was not instigated as it does not improve survival in asymptomatic animals.^43^ In symptomatic dogs, options include artificial pacemaker implantation and medical management with anticholinergics^1^ and cilostazole.^44^ Were general anaesthesia not declined, premedication with a parasympatholytic agent, such as atropine, could have been attempted to abolish excessive vagal tone or, in the absence of a response, temporary pacing used to maintain an acceptable heart rate, cardiac output and mean arterial pressure. Drugs that lead to marked bradycardia, such as alpha-2 agonists, should be avoided and opioids that may reduce heart rate even at low doses^45^ should be used judiciously.

Cats with increased vagal tone have an excellent prognosis, whereas in animals with SND, the short-term prognosis is often good but there is disease progression. Common sequalae in dogs and humans are associated with congestive heart failure (CHF) and thromboembolic disease is reported in humans.^1,21^ A retrospective analysis of outcomes in dogs found a mean survival time of 18 months with no difference between symptomatic and asymptomatic patients, or symptomatic cases with or without treatment.^1^ The mean age at diagnosis in dogs was 11 ± 3.0 years, and the incidence of cardiac death higher in clinically affected animals despite similar numbers developing CHF. Some dogs survived several years after diagnosis, suggesting that other age-related diseases contributed to their deaths, although these may be associated with poor cardiac output compromising organ function.^1^

## Conclusions

Although unusual, this case highlights the importance of pre-anaesthetic examination in identifying unexpected abnormalities in apparently healthy young patients. It also raises awareness of SND as a differential diagnosis in cats presenting with bradyarrhythmia. Careful examination of the cardiovascular system in animals undergoing routine neutering procedures, combined with a reduction in sympathetic stimulation by designing a low-stress clinic environment, may lead to more frequent recognition.
